# The MicroRNA miR-155 Is Essential in Fibrosis

**DOI:** 10.3390/ncrna5010023

**Published:** 2019-03-12

**Authors:** Mousa G. Eissa, Carol M. Artlett

**Affiliations:** Department of Microbiology & Immunology, Drexel University College of Medicine, Drexel University, 2900 Queen Lane, Philadelphia, PA 19129, USA; mosa1034@gmail.com

**Keywords:** miR-155, fibrosis, inflammasome, fibroblasts, TGF-β1

## Abstract

The function of microRNAs (miRNAs) during fibrosis and the downstream regulation of gene expression by these miRNAs have become of great biological interest. miR-155 is consistently upregulated in fibrotic disorders, and its ablation downregulates collagen synthesis. Studies demonstrate the integral role of miR-155 in fibrosis, as it mediates TGF-β1 signaling to drive collagen synthesis. In this review, we summarize recent findings on the association between miR-155 and fibrotic disorders. We discuss the cross-signaling between macrophages and fibroblasts that orchestrates the upregulation of collagen synthesis mediated by miR-155. As miR-155 is involved in the activation of the innate and adaptive immune systems, specific targeting of miR-155 in pathologic cells that make excessive collagen could be a viable option before the depletion of miR-155 becomes an attractive antifibrotic approach.

## 1. Introduction

MicroRNAs (miRNAs) are important regulators of gene expression that control not only the fate of a cell but also the corresponding cell’s response to stimuli. Specific miRNAs are involved in fibrosis [1,2,3]; however, extraordinarily little is known about how these miRNAs are regulated or how they regulate the fibrotic axis. MiRNA-155 (miR-155) is a multifunctional microRNA initially identified as having immune regulatory functions, as it plays a critical role in innate and adaptive immune responses [4,5,6,7]. Recent evidence suggests that miR-155 is also crucial for the generation of fibrosis [8,9,10]. MiR-155 is overexpressed in fibrotic tissues, and the deletion of miR-155 abrogates fibrosis. These fundamental observations signify that miR-155 is integral to the laying down of collagen and mediates TGF-β1 signaling in this pathologic setting [11,12,13,14]. This review highlights the role of miR-155 and how it facilitates downstream signaling to drive fibrosis. 

## 2. Fibrosis Is a Common Pathology in Many Diseases

Fibrosis is tightly associated with excessive collagen synthesis, primarily, types I and III collagens (COL1A1 and COL3A1, respectively), and with the production of other extracellular matrix proteins (e.g., fibronectin, fibrillin, elastin, laminin) by fibroblasts. However, other cells such as macrophages can synthesize collagens during fibrosis [15,16]. Fibrosis is a chronic progressing pathology that causes scarring of tissues and can severely affect the function of an organ leading to significant morbidity and mortality. We do not understand all the mechanism(s) that drive fibrosis, but this disorder can occur in idiopathic pathologies such as pulmonary fibrosis or can be associated with a diverse spectrum of chronic diseases including systemic sclerosis [17,18], rheumatoid arthritis [19,20], chronic hepatitis infections [21,22], etc. Once started, there is no effective therapeutic that halts the progression of fibrosis. It has been estimated that approximately 45% of people in the Western world will die of some type of fibrotic disease [23]. While little is known about the pathology of fibrotic disorders, researchers have started to elucidate what regulates the expression of collagen via miRNAs [8,9,10,24,25,26].

Mir-155 is upregulated during inflammation and, in the context of cancer, it regulates the tumor inflammatory cytokine microenvironment by targeting the post-transcriptional regulation of C/EBPβ [27]. The causal relationship between inflammation and cancer is now widely accepted [28], and the role of cancer-associated fibroblasts in this setting is known [29,30,31]. In the context of cancer, cancer-associated fibroblasts drive the synthesis of excessive extracellular matrix (ECM). This matrix mediates the response of the tumor to chemotherapeutics not only by further upregulating collagen synthesis but also by impeding the response of the tumor to therapy [32]. Indeed, the same cell phenotypes and soluble and matrix components involved in wound healing also promote fibrosis and tumor progression. For example, miR-155 orchestrates cytokines’ responses in cancer-associated macrophages [27]. miR-155 appears to be critical to the fibrotic process, as it has been found to be elevated in fibrotic tissue and when depleted in in vivo models, the lack of miR-155 abrogates fibrosis [8,9,10,26].

## 3. The Downstream Consequences of Mir-155 Upregulation

Mir-155 is encoded in the *BIC* gene [33]. It was the first identified oncomiR to be upregulated by different oncogenic viruses [34]. Some oncogenic viruses also encode orthologs of miR-155 [35]. The integration of retroviruses into *BIC* led to the overexpression of miR-155 in B cells, causing B cell tumors [36]. Highlighting this, Bolisetty et al. [34], identified two different B cell lymphoma lines infected with the reticuloendotheliosis virus strain T that overexpressed miR-155. They also observed increased miR-155 in chicken embryonic fibroblasts infected with the reticuloendotheliosis virus strain T. The reticuloendotheliosis virus strain T encodes the *v-rel* oncogene [37], and this oncogene has similar homology to the check-point oncogene *c-rel* [38]. Like c-Rel [39], v-Rel upregulates the expression of the AP-1 transcription factor which then enhances the expression of miR-155 [40,41,42,43]. The activation of the B cell receptor also leads to the upregulation of miR-155 expression via AP-1 [43]. which points to a common regulatory pathway for this oncomiR.

miR-155 can be upregulated in various circumstances, including cancer [23,24,25,26], viral infections [43,44,45], and during immune activation [4,5,46,47]. Multiple gene targets have been identified for miR-155 in numerous different cell types [4,24,29,30,31,32]. Inflammatory cytokines that upregulate miR-155 are IL-1β [13], TGF-β1 [14], TNF-α [15,16,17], and INF-γ [17,18]. miR-155 can be induced by other proinflammatory stimuli such as lipopolysaccharide that activates Toll-like receptor 4 on macrophages and dendritic cells [41,48] and by bleomycin, as discussed below. In contrast, IL-10 is a potent inhibitor of miR-155 [19]. IL-2 and IL-15 also upregulate the expression of miR-155. This upregulation occurs via signal transducers and activator of transcription (STAT)-5 [49], as well as through other STATs such as STAT-1 and STAT-3. Janus kinases (JAKs) also upregulate miR-155 expression, and the blockade of JAK signaling abrogates miR-155 [15]. miR-155 is involved in this signaling pathway and lowers the expression of suppressor of cytokine signaling (SOCS)-1 protein. SOCS-1 is a negative regulator of JAK/STAT signaling [50], and in its absence, miR-155 is able to promote its own upregulation.

## 4. Macrophage-Derived Exosomes Carrying Mir-155 Mediate Fibrosis

Exosomes are cell-derived vesicles produced by many different cell types that function in signaling between cells. Exosomes carry a variety of different cargoes, such as cytokines and microRNAs. miR-155 appears to be an important microRNA consistently carried in exosomes and transferred to other cells [51]. The transfer of miR-155 from a cell occurs during malignancy [52] and in fibrosis [53]. M2 macrophages releasing miR-155-containing exosomes mediate fibroblast inflammation during cardiac injury, in addition to suppressing fibroblast proliferation [53]. Macrophages are capable of secreting excessive amounts of collagen, contributing to fibrotic process seen in tissues [15,16]. Intriguingly, exosomes derived from angiotensin II-stimulated macrophages induced miR-155 expression in cardiac fibroblasts, but the direct culturing of cardiac fibroblasts with angiotensin II did not induce miR-155 in these cells. To confirm this observation, Wang et al. [53] depleted exosomes from macrophage-conditioned media, and this reduced the expression of miR-155 in fibroblasts. In contrast, in miR-155-deficient cardiac fibroblasts, exosomes carrying miR-155 induced the expression of miR-155. This suggests that cardiac fibroblasts do not endogenously express their own miR-155 in response to angiotensin II, but exosomes derived from macrophages stimulated with angiotensin II can transfer miR-155 to cardiac fibroblasts [53] to orchestrate downstream signaling events that drive fibrosis in these cells.

Fibroblast cell lines derived from explants from patients with systemic sclerosis (SSc) continue to maintain a high level of collagen expression in culture even in the absence of macrophages [8,54,55,56]. Normal fibroblasts cell lines can be stimulated with bleomycin to induce collagens. In contrast to these studies, we found that bleomycin induced a 4–6 fold increase in miR-155 expression in C57BL/6 wild-type lung fibroblasts [8]. We used cell lines established from lung explants that had been cultured for several generations prior to testing. Therefore, the chance of contaminating macrophages present in the cell line mentioned above was slim. Thus, we believe that in the right setting, miR-155 is upregulated in fibroblasts in the absence of macrophages.

## 5. Mir-155 in Wound Healing and Skin Fibrosis

Many cells contribute to the process of wound healing and stimulate the process of deposition of ECM proteins. In addition to fibroblasts and myofibroblasts, tissue-resident macrophages also play a crucial role. Macrophages promote inflammation, fibroblast proliferation, and matrix regeneration within a wounded tissue. Together with macrophages, fibroblasts responding to macrophage signaling, secrete collagens and other extracellular matrix proteins and help to promote angiogenesis and re-epithelialization. miR-155 is upregulated during this inflammatory phase of healing corresponding to the influx of inflammatory cells, but evidence now shows that miR-155 is upregulated in fibroblasts (directly or indirectly) and is involved in ECM deposition in later phases of wound healing.

In our laboratory, we have defined the role for miR-155 in dermal fibrosis. We reported that IL-1β appears to be a central cytokine driving fibrosis, because blockade of capase-1, the enzyme that activates IL-1β, abrogated fibrosis. Because IL-1β upregulated miR-155 [9] and because the inflammasome was activated in SSc fibroblasts, we sought to determine whether miR-155 was elevated in SSc fibroblasts [54]. In keeping with an activated IL-1β signaling pathway in SSc fibroblasts, we found miR-155 to be overexpressed in these cells (both in lung-derived and dermal fibroblasts). This confirms the observations of Christmann et al. [57], who also found upregulated miR-155 expression in SSc lungs. We further found that miR-155 expression in SSc fibroblasts was dependent on the activation of the inflammasome, because inhibition of the inflammasome signaling cascade using a caspase-1 inhibitor (YVAD) abolished miR-155 expression. Further confirming the crucial role of miR-155 expression, we showed for the first time that fibroblasts derived from NLRP3-deficient mice could not induce miR-155 expression when stimulated with bleomycin. We therefore believe that, in response to bleomycin, the NLRP3 inflammasome is required for miR-155 expression and we speculate that this occurrs via inflammasome-mediated processing of IL-1β [8]. 

We then explored if miR-155 directly participated in fibrosis. We found that bleomycin could not induce collagen synthesis in fibroblasts derived from mice devoid of miR-155, whereas, the restoration of miR-155 using lentiviral transduction caused the upregulation of collagen in an IL-1-dependent manner. In fibroblasts, IL-1β induced miR-155 expression and correspondingly increased the synthesis of collagen in miR-155-replete fibroblasts, and this effect was abolished by IL-1RA [8]. Overall, this suggests that miR-155 may be an essential regulator in SSc fibrosis, and its expression is downstream of inflammasome activation, requiring IL-1β.

Mir-155 appears to be a critical regulator of wound healing and fibrosis. Our studies demonstrated that in fibrosis, miR-155 expression requires the activation of the NLRP3 inflammasome. Thus, we propose that the NLRP3 inflammasome is the initiator causing the upregulation of IL-1β transcription and autocrine signaling that drives the expression of miR-155 [8]. We further found that miR-155 synergized with the NLRP3 inflammasome in driving a positive feed-forward IL-1β-mediated signaling mechanism that further promoted the release of IL-1β and the expression of miR-155. This then caused further autocrine signaling leading to continual collagen expression. Inhibiting IL-1β expression either by blocking its receptor with the IL-1 receptor antagonist or by inhibiting inflammasome-mediated activation of caspase-1 broke this feed-forward cycle and abrogated miR-155 expression. As a result, reduced collagen synthesis also occurred in pathologically fibrotic fibroblasts [8].

In support of our findings, Wu et al. [58] found that the downregulation of miR-155 expression caused reduced collagen synthesis. They studied changes in SH2 domain-containing iniositol-5-phosphatase 1 (SHIP1) and observed that the expression of SHIP1 was negatively correlated with miR-155 levels [58]. SHIP1 is essential not only for collagen production but also for fibroblasts’ proliferation, survival, and migration [59,60,61]. In addition to this observation, suppression of the phosphatidylinositol-4,5-bisphosphate 3-kinase (PI3K)/Akt pathway occurred when miR-155 was downregulated. The PI3K/Akt pathway is involved in cell metabolism, cell survival, and protein synthesis [62,63,64,65], all processes observed in fibrosis. Furthermore, in a separate study, Yang et al. [58] found that by downregulating the expression of miR-155, fibroblasts had reduced proliferation. Yang et al. also investigated cross-signaling between macrophages and fibroblasts, because macrophages play a prominent role in driving fibrosis. Using anti-sense miR-155 oligonucleotides to reduce miR-155 expression in macrophages, they demonstrated a significant reduction in macrophage TGF-β1 and IL-1β expression, whereas TNF-α and IL-6 remained unchanged [58]. 

In quiescent fibroblasts, the downregulation of miR-155 produced no effect on the expression of COL1A1 or COL3A1, suggesting that in non-fibrotic fibroblasts, collagen expression is not dependent on miR-155 [58]. This observation is also in keeping with our laboratory’s results, as we observed that collagen expression in quiescent fibroblasts was not dependent on inflammasome activation or IL-1β [54]. Overall, these observations are not surprising, as activation of the wound healing mechanism is only needed in the presence of tissue damage. However, understanding the fundamental differences in collagen synthesis between quiescent fibroblasts and activated fibroblasts/myofibroblasts is paramount to the development of effective antifibrotics. These observations have yielded some clues towards this end.

Supporting the above observations, van Solingen et al. [66] showed that during wound healing, the absence of miR-155 promoted better healing than observed in its presence. Wound healing is a well-orchestrated process that involves a balance between pro-inflammatory and endogenous anti-inflammatory mechanisms. When these mechanisms are correctly balanced, tissue inflammation is limited, and wound healing is promoted [67]. van Solinger et al. [66], studied wound healing in miR-155-deficient mice over 10 days. They observed decreased granulation and faster formation of de novo hair follicles than in wild-type mice, suggesting accelerated wound healing. Intriguingly, miR-155-deficient mice presented a high number of macrophages in their wounds. van Solingen [66] reported that miR-155 attenuated the expression of FIZZ1 in M2 macrophages but had no effect on FIZZ1 expression in M1 polarized macrophages [66]. FIZZ1 (found in inflammatory zone 1) is induced by the profibrotic molecule bleomycin and is a crucial profibrotic mediator. FIZZ1 promotes COL1A1 and α-smooth muscle actin (α-SMA) expression in lung fibroblasts [68]. Whether the loss of miR-155 decreases FIZZ1 expression in fibroblasts and whether there is reduced COL1A1 and α-SMA as a result, remain unknown.

Ye et al. [69] also found that the inhibition of miR-155 expression promoted better wound healing in diabetic rats. The Ye laboratory reported that the reduced expression of miR-155 promoted “new well-formed granulation tissue with marked proliferation of fibroblasts and new, well-formed capillaries” [69]. Intriguingly, in the diabetic miR-155 antagomir cohort, they observed decreased numbers of macrophages. Ye [69] suggested that miR-155 aided in the recruitment of inflammatory cells into the diabetic wound bed. 

Mir-155-overexpressing fibroblasts showed increased migration through collagen gels [9]. This suggests that miR-155 could help to promote wound closure by causing fibroblasts to migrate faster into damaged tissues. Furthermore, Yan et al. [10] found that miR-155 regulated the Akt and Wnt/β-catenin pathways in fibrotic fibroblasts derived from patients with SSc. Additional studies on the functional role of miR-155 in SSc also demonstrated elevated miR-155 in SSc fibroblasts [10]; however, this study did not explore what caused the increase in miR-155 but investigated the downstream responses mediated by miR-155. Intriguingly Yan et al. [10], found that patients with the milder sclerotic variant, morphea, had higher levels of miR-155 than patients affected by the more severe systemic form of SSc fibrosis but they did not discuss this observation. Yan et al. [10] also observed that targeting miR-155 caused the suppression of β-catenin and reduced the levels of phosphorylated Akt. This occurred by miR-155-dependent downregulation of CK1α and SHIP1 in the presence of TGF-β1. The development of fibrosis requires both β-catenin and the phosphorylation of Akt, and these pathways are consistently upregulated in other fibrotic diseases [9,70,71]. Pottier et al. [33] showed that miR-155 could significantly decrease the release of keratinocyte growth factor that had been induced by IL-1β or TNF-α in human normal pulmonary fibroblasts and demonstrated that it played a role in epithelial–mesenchymal transition. Therefore, miR-155 may have an important pathophysiological impact during acute lung injury or pulmonary diseases [34].

## 6. The Functional Role of Mir-155 in Cardiac Fibrosis and Hypertrophy

There is ubiquitous expression of miR-155 in the cardiac muscle [72]. miR-155 deficiency attenuated myocardial damage mediated by diabetes [73]. Considering the high-glucose environment, collagen deposition by cardiac fibroblasts was enhanced, and this was mediated by miR-155, such that depletion of miR-155 mitigated this effect [73]. The high-glucose environment induced the expression of TGF-β1 and Smad2 and increased Smad2 phosphorylation. In addition, the ratio between Smad2 and the levels of phosphorylated Smad2 increased over time, suggesting greater activation of Smad2. This occurred in a miR-155-dependent manner [73]. In contrast, Smad3, another regulatory Smad involved in TGF-β1 signaling, remained unchanged [11].

Further evidence of the role for miR-155 in cardiac fibrosis came from a study suggesting that miR-155 is necessary for the expression of COL1A1 and COL3A1 [26]. In addition, the depletion of miR-155 in this study downregulated the expression of α-SMA (a marker of activated fibroblasts/myofibroblasts) and suppressed cell proliferation [26].

*c-Ski* is a proto-oncogene that is a negative regulator of TGF-β1 signaling. c-Ski directly interacts with Smad2, Smad3, and Smad4 on the TGF-β1-responsive promoter element and represses the ability of these proteins to activate transcription [12]. TGF-β1 gradually reduced c-Ski in a dose- and time-dependent manner [14]. At the same time, in the presence of TGF-β1, the reduced c-Ski levels helped to drive endothelial–mesenchymal transition, whereas the over expression of c-Ski caused the blockade of this process. With increased c-Ski, there was a decrease of fibrotic markers such as collagen, α-SMA, vimentin, snail, slug, and twist [13,14]. Wang et al. [13,14] further demonstrated that this mechanism occurred via miR-155 binding to the 3’ UTR of *c-Ski*, suggesting that miR-155 plays an integral role in fibrotic pathologies by mediating TGF-β1 signaling via a c-Ski-regulated mechanism.

Another pro-fibrotic molecule is angiotensin II. Angiotensin II functions to stimulate the TGF-β1 pathway and induces collagen [74,75,76]. Infused angiotensin II increased cardiac remodeling in mice and also induced hypertrophy in both miR-155-deficient mice and wild-type mice compared to sham-treated mice. However, the wild-type mice had significantly enlarged hearts compared to the miR-155-deficient mice [72]. Upon angiotensin II infusion, the resulting inflammatory cell infiltration into the cardiac muscle in the miR-155-deficient mice was reduced, and collagen and α-SMA expression also decreased [72]. Wei et al. [72] reported that forced expression of miR-155 induced α-SMA, further supporting the notion that miR-155 drives fibroblast to myofibroblast differentiation. In the absence of miR-155, angiotensin II elevates SOCS1 (suppressor of cytokine signaling-1) expression. Wei et al. [72] then suggested that SOCS1 might be involved in the generation of miR-155 cardiac fibrosis. They further found that miR-155-deficient mice had weakened TGF-β1/Smad3 signaling compared to their wild-type counterparts, signifying that miR-155 promotes fibrosis via the TGF-β1/Smad3 pathways, and this involved SOCS1 [72].

An additional study on the role of miR-155 in cardiac hypertrophy was published by Seok et al. [77]. These authors reported the role for JARID2 in mediating miR-155 signaling in cardiomyocytes. JARID2 (Jumonji- and AT-rich interaction domain (ARID)-domain-containing protein-2) is a DNA-binding protein that functions as a transcriptional repressor. Seok et al. [77] found that the inhibition of JARID2 partially rescued the hypertrophic growth, and this rescue could be inhibited by the depletion of miR-155. Norfo et al. [78] then found additional evidence for the role of miR-155 in JARID2 signaling in primary myelofibrosis. They observed a negative correlation between JARID2 and miR-155, suggesting that the loss of JARID2 expression mediated by miR-155 drives fibrosis in the bone marrow [78].

## 7. Mir-155 and the Fibrotic Liver

MiR-155 has been extensively studied in the context of liver fibrosis. Steatosis is one of the first pathologic responses by the liver due to binge or chronic alcohol consumption. This causes the accumulation of lipids in the liver that leads to lipid superoxidation, oxidative stress, apoptosis, and hepatic inflammation [79]. Alcohol-induced macrophage activation occurs via a miR-155-dependent mechanism, as miR-155 targets genes that are involved in lipid metabolism and early fibrosis [80]. MiR-155 showed increased expression in macrophages and hepatocytes, although, considering the publication by Wang et al. [53], one wonders whether hepatocytes truly expressed miR-155 or if miR-155 was delivered from macrophages to the hepatocytes via exosomes. Some evidence suggests that miR-155 is only produced by mononuclear cells and not by hepatocytes [81], but other studies report that hepatocytes do express miR-155 [82,83].

Bala et al. [81] found depletion of miR-155 significantly reduced alcohol-induced liver injury. They further found that the reduction in fat accumulation in the liver correlated with an increased expression of peroxisome proliferator-activated receptor response element and peroxisome proliferator-activated receptor-α. Furthermore, miR-155-deficient animals demonstrated abrogated fibrosis in the liver [81]. miR-155-deficient mice had reduced alcohol-induced hepatic inflammation and suppressed liver macrophage and neutrophil numbers. Intriguingly, macrophage polarization was skewed by alcohol towards the M2 (pro-fibrotic) phenotype, and this required TLR4. Bala et al. [81] found that miR-155 drove macrophage polarization by targeting C/EBPβ. The absence of miR-155 prevented M2 polarization [81]. The expression of C/EBPβ was only observed in macrophages derived from miR-155-deficient animals, and this corresponded to increased STAT3. Further considering the depletion of miR-155 in liver disease, miR-155-deficient mice had reduced TGF-β1, collagens, and vimentin on exposure to ethanol [81].

Elegant studies by Blaya et al. [81] also demonstrated that the restoration of miR-155 expression in inflammatory cells directly affected liver injury in miR-155-deficient mice [84]. miR-155 deficient mice transplanted with wild-type bone marrow (miR-155-replete) were stimulated with concanavalin A to cause liver fibrosis. After hematopoietic reconstitution, miR-155-deficient mice transplanted with wild-type bone marrow had partial rescue of the phenotype. These mice demonstrated acute liver injury, and this suggested that miR-155 expression in inflammatory cells caused liver inflammation and damage [84]. Considering this, Blaya et al. [81] studied the expression of miR-155 in liver and circulating peripheral blood mononuclear cells from patients with autoimmune hepatitis, alcoholic liver disease, and nonalcoholic steatohepatitis. They found that in the three cohorts, all patients had reduced PBMC miR-155 levels, whereas they noted significantly elevated miR-155 only in the liver samples derived from decompensated cirrhosis and autoimmune hepatitis [84].

The methionine–choline-deficient diet is used to induce steatohepatitis. This diet promoted the expression of miR-155 in both parenchymal and non-parenchymal cells. This diet also caused lipid accumulation, inflammation, and fibrosis in the liver. Inflammation is an important feature of liver damage that precedes liver fibrosis. In keeping with the observations reported above, the expression of miR-155 was abundant in immune cells but was low in hepatocytes [80]. In other models, miR-155 deficiency attenuated steatosis and fibrosis; however, curiously, Csak et al. found that fibrosis induced by the methionine–choline-deficient diet occurred by a mechanism that did not reduce inflammation when miR-155 was depleted [80]. Csak et al. [80] then investigated functional signaling in the liver to try to determine the mechanism behind the abrogation of fibrosis when there was no reduction in inflammation in miR-155-deficient mice. They demonstrated that liver miR-155 expression was dependent on TNF-α, and intriguingly, TNF-α levels were higher in miR-155-deficient animals. In addition, monocyte chemoattractant protein-1 had similar expression levels in the two cohorts, but IL-1β was reduced in the miR-155-deficient mice [80]. They also showed that there was no difference in TGF-β1 levels but found platelet-derived growth factor to be suppressed in the miR-155-deficient mice. They then evaluated TGF-β1-downstream signaling molecules and found Smad2 protein to be unchanged. whereas the levels of Smad3 and vimentin proteins were significantly lower [80].

## 8. Mir-155 in Diagnostics, Screening, and Treatment

The serum levels of miR-155 correlate with radiological and forced vital capacity levels suggesting that this miRNA could be used to predict disease severity in patients with pulmonary fibrosis [85,86]. Therefore, miR-155 could be used in a screening platform for fibrotic pathologies. However, this finding was not replicated in hepatic fibrosis, where miR-155 was not associated with the degree of fibrotic damage [87]. Mir-155 is significantly upregulated during heart failure and could be used (in conjunction with a panel of other miRNAs) as a diagnostic marker for this pathology [88]. However, to date, this screening modality has not been developed nor FDA-approved. Therapeutically targeting miR-155 has shown promise in an animal model of wound heaing. Yang et al. [89] delivered a miR-155 antagomir into a wound edge and found that there was a reduced influx of inflammatory cells and reduced levels of proinflammatory cytokines such as TNF-α and IL-1β. In addition, IL-10 levels were increased. They also found ECM proteins to be decreased, including Col1, Col3, and α-Sma. In a follow-up paper, Yan et al. [10] also used an antagomir for miR-155 to inhibit experimental fibrosis in a mouse model of SSc. Overall, these findings are provocative and suggest that the delivery of a miR-155 antagomir could ameliorate fibrosis.

## 9. Conclusions

In this review, we have summarized the role of miR-155 in fibrosis. MiR-155 is a crucial microRNA for the development of fibrosis. Depletion of miR-155 abrogates collagen synthesis in both animal models of fibrosis and human fibroblast cell lines derived from fibrotic lesions. Studies also show that miR-155 is crucial in mediating TGF-β1 activation in fibroblasts and macrophages and that it might provide an alternative target to treat fibrotic pathologies. Inhibiting TGF-β1 in fibrosis was not as successful in clinical trials as anticipated [90]. This could be because additional profibrotic molecules can upregulate miR-155, e.g., angiotensin II, and therefore drive fibrosis independently of TGF-β1. Specifically targeting miR-155 in pathologic cells that drive fibrosis using antagomirs seems an attractive approach for the control of collagen deposition in fibrotic diseases.
